# Enhancing docosahexaenoic acid production of *Schizochytrium* sp. by optimizing fermentation using central composite design

**DOI:** 10.1186/s12896-022-00769-z

**Published:** 2022-12-09

**Authors:** Jun Ding, Zilin Fu, Yingkun Zhu, Junhao He, Lu Ma, Dengpan Bu

**Affiliations:** State Key Laboratory of Animal Nutrition, Institute of Animal Sciences, Chinese Academy of Agricultural Sciences, No. 2 Yuanmingyuan West Road, Beijing, 100193 China

**Keywords:** Center composite design, DHA, Plackett–Burman design, *Schizochytrium* sp.

## Abstract

Docosahexaenoic acid (DHA) can improve human and animal health, particularly including anti-inflammatory, antioxidant, anticancer, neurological, and visual functions. *Schizochytrium* sp. is a marine heterotrophic protist producing oil with high DHA content, which is widely used in animal and food production. However, different fermentation conditions have intensive impacts on the growth and DHA content of *Schizochytrium* sp. Thus, this study aimed to enhance the DHA yield and concentration of *Schizochytrium* sp. I-F-9 by optimizing the fermentation medium. First, a single-factor design was conducted to select a target carbon and nitrogen source from several generic sources (glucose, sucrose, glycerol, maltose, corn syrup, yeast extract, urea, peptone, and ammonium sulfate). The Plackett–Burman design and the central composite design (CCD) were utilized to optimize the fermentation mediums. *Schizochytrium* sp. in 50-mL fermentation broth was cultured in a 250 mL shake flask at 28 °C and 200 rpm for 120 h before collecting the cell pellet. Subsequently, the cell walls were destroyed with hydrochloric acid to extract the fatty acid using n-hexane. The DHA content was detected by gas chromatography. The single-factor test indicated that glucose and peptone, respectively, significantly improved the DHA content of *Schizochytrium* sp. compared to the other carbon and nitrogen sources. Glucose, sodium glutamate, and sea crystal were the key factors affecting DHA production in the Plackett–Burman test (*P* = 0.0247). The CCD result showed that DHA production was elevated by 34.73% compared with the initial yield (from 6.18 ± 0.063 to 8.33 ± 0.052 g/L). Therefore, the results of this study demonstrated an efficient strategy to increase the yield and content of DHA of *Schizochytrium* sp.

**Supplementary Information:**

The online version contains supplementary material available at 10.1186/s12896-022-00769-z.

## Introduction

The beneficial effects of docosahexaenoic acid (C22:6 n-3; DHA) have been extensively and systematically explored in humans and animals for decades [1]. According to by Zhang and Spite [2] and Zhang et al. [3], DHA can regulate inflammation, oxidative stress, immunity and cholesterol metabolism, which can efficiently prevent cancer, diabetes and thrombosis. In addition, as a long-chain unsaturated fatty acid, DHA is an essential substrate of phospholipids, triglycerides and some free fatty acids in vertebrate animals. Thus, DHA plays an important role in human and animal health [4]. The rapidly increasing requirement for DHA worldwide has intensified the demand for DHA production [5].

The main sources of DHA is seafood, mainly including fish and algae [6]. DHA yield from fish oil has been limited due to the increasing environmental and food safety concerns, such as ecological diversity maintaining and heavy metal pollution, which leads to insufficient production of DHA to meet the growing demands [7, 8]. Therefore, *Schizochytrium* sp. has been developed as alternative sources for DHA production [9, 10]. In 1964, Goldstein and Belsky isolated *Schizochytrium* sp. from Long Island Sound and classified it to Thraustochytriaceae [11]. Subsequently, many studies [12] have confirmed that *Schizochytrium* sp. is one of the most commercially attractive and valuable sources of DHA [13] and a heterotrophic unicellular strain that can be safely used as dietary supplement [7, 14]. Clinical trials have shown that the bioactivities of microbial-derived DHA can be comparable to that from fish oil in reducing plasma triglycerides, promoting redox properties, and protecting cardiovascular systems [8, 15]. Compared with other marine heterotrophic protists, *Schizochytrium* sp. is more potential in DHA production with high lipid concentration accounting for 36–84% of biomass, in which the DHA concentration exceeds 62% of the total lipid [13, 16, 17].

As *Schizochytrium* sp. has more advantages than fish oil, extensive studies have been conducted to promote the DHA biosynthesis of *Schizochytrium* sp. [18] using mutagenesis screening, adaptive evolution, multi-omics technologies, and metabolic engineering methods [13]. Furthermore, multiple studies have focused on the optimization of the fermentation process to improve DHA production and biomass, including improving the nutritional conditions [19] (carbon, nitrogen and exogenous additives) and growth conditions [20, 21] (osmotic pressure, dissolved oxygen (DO), pH and aeration). For use in animal production, a *Schizochytrium* strain should have high biomass with efficient capability of lipid and preferably DHA accumulation. Therefore, it is necessary to optimize the culture conditions to maximize biomass and DHA yield. Fu et al. [22] obtained DHA-rich *Schizochytrium* sp. S1 by mutagenesis and then carried out the optimization of fermentation to improve the DHA yield of *Schizochytrium* sp. S1 from 5.41 to 6.52 g/L. Zhao et al. [23] obtained a strain with high DHA by atmospheric and room temperature plasma (ARTP) mutagenesis combined with malonic acid chemical screening. Then, they used an optimized culture strategy to increase the DHA production by 1.8-fold. Because efficient microbial-derived DHA production depends on the growth period, the composition of the medium and the mode of fermentation, it is thus essential that each new strain of *Schizochytrium* sp. should be optimized for individual culture conditions. For efficient microbial-derived DHA production, the suitable fermentation conditions for DHA yield by the *Schizochytrium* sp. I-F-9 were investigated. The influence of the fermentation medium in DHA production was investigated by a single-factor experimental design in conjunction with a central composite experimental design.

## Materials and methods

### Experimental design

**Fig. 1:**
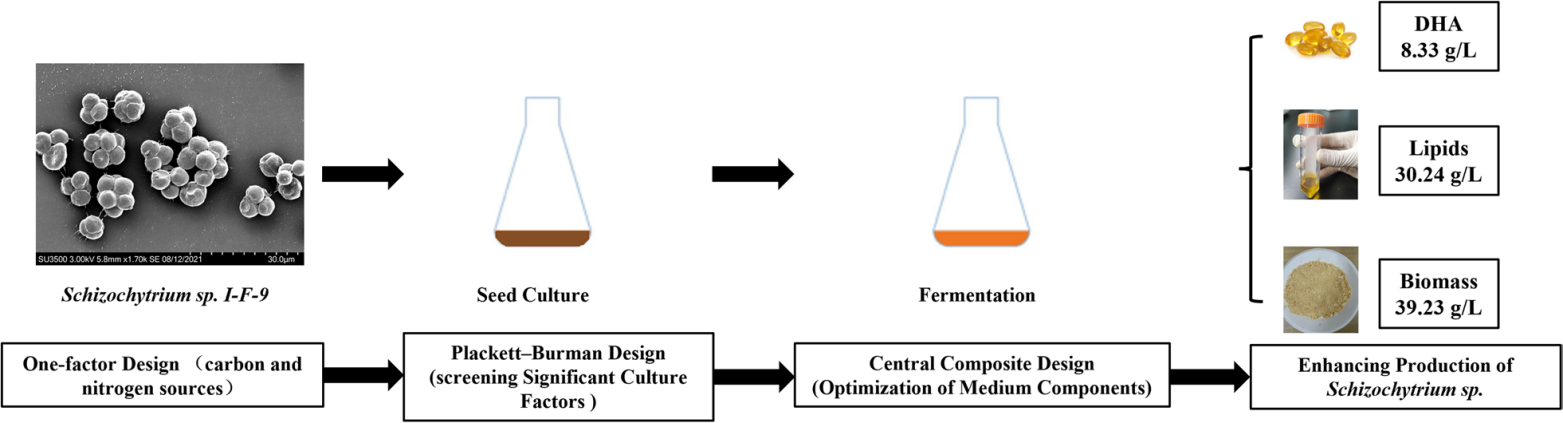
Experimental design process

This study determined the values of critical process parameters affecting the DHA production in *Schizochytrium* sp. utilizing sodium glutamate as the main stimulator. The fermentation process and experimental design are shown in Fig. 1. First, the best carbon and nitrogen medium affecting the fermentation of *Schizochytrium* sp. were determined from common carbon and nitrogen sources using a single-factor experiment. Under shake-flask fermentation conditions, 100 g/L glucose, sucrose, glycerol, and maltose as carbon sources and 10 g/L corn steep liquor, yeast extract, urea, peptone, and ammonium sulfate as nitrogen sources were used for fermentation to screen the best carbon and nitrogen sources (other conditions remained unchanged). Each trial was set up with three replicates.

The optimal impact factor was then decided by a sequence of experiments utilizing a Plackett–Burman design (Design-Expert version 11.0.0). The evaluated factors were as follows: glucose, peptone, sodium glutamate, KH_2_PO_4_, MgSO_4_ 7H_2_O, and sea salt. Each independent variable was tested at a low (−) and a high (+) level. The low levels of a variable were taken as the current fermentation conditions. The high level was 1.25 times the low level. Table 1 demonstrates a series of the analyzed factors, their values and corresponding levels. The 12 tests output by the Design-Expert software, and are listed in Table 2. The effects of each factor (A–F), the significant value (*P*-value), and the F-value (F-test results) were presented in this study. When *P* < 0.05, the factor was considered as the most significant parameter to influence DHA production.

**Table 1:** Variables range of Plackett–Burman design

Variable code	Variable	Unit	Low (− 1)	High (+ 1)
A	Peptone	g/L	5.6	7
B	Glucose	g/L	100	125
C	Sodium Glutamate	g/L	20	25
D	MgSO_4_ 7H_2_O	g/L	7.2	9
E	KH_2_PO_4_	g/L	2.5	3.125
F	Sea Salt	g/L	34.4	43
G–K	Dummy variable	–	–	–

**Table 2:** Plackett–Burman design of the experiments

Run	A: peptone (g/L)	B: glucose (g/L)	C: sodium glutamate (g/L)	D: MgSO_4_ 7H_2_O (g/L)	E: KH_2_PO_4_ (g/L)	F: sea salt (g/L)
1	7	125	20	9	3.125	18.75
2	7	125	20	7.2	2.5	18.75
3	5.6	100	20	9	2.5	18.75
4	5.6	100	25	7.2	3.125	18.75
5	5.6	125	25	9	2.5	15
6	7	100	25	9	3.125	15
7	5.6	125	20	9	3.125	15
8	7	125	25	7.2	2.5	15
9	7	100	20	7.2	3.125	15
10	5.6	100	20	7.2	2.5	15
11	7	100	25	9	2.5	18.75
12	5.6	125	25	7.2	3.125	18.75

After screening for the most significant factors that influence the DHA content, the central composite design (CCD) was utilized to determine the parameter values that resulted in the optimal DHA yield. The Design-Expert software generated this process to table a list of experiments. The CCD design involved five coded values: − 2, − 1, 0, 1, and 2; a trial design was established using the central and axial points (Table 3). Based on the results of the Plackett–Burman design test, the insignificant factors were maintained at the low level in the CCD experiment. Six replications of the central point were employed (Table 4). The trial results of the CCD design were fitted with a quadratic polynomial equation by multiple regression modeling utilizing the Design-Expert software, and the optimal point was predicted.

**Table 3:** Variables range of CCD experiments

Variable	Unit	Level				
		− 2	− 1	0	1	2
Glucose	g/L	91.48	100	112.5	125	133.52
Sodium glutamate	g/L	18.30	20	22.5	25	26.7
Sea salt	g/L	13.72	15	16.88	18.75	20.03

**Table 4:** CCD of the experiments

Run	Glucose (g/L)	Sodium glutamate (g/L)	Sea salt (g/L)
1	100	20	15
2	125	20	15
3	100	25	15
4	125	25	15
5	100	20	18.75
6	125	20	18.75
7	100	25	18.75
8	125	25	18.75
9	91.48	22.5	16.88
10	133.52	22.5	16.88
11	112.5	18.30	16.88
12	112.5	26.70	16.88
13	112.5	22.5	13.728
14	112.5	22.5	20.03
15	112.5	22.5	16.88
16	112.5	22.5	16.88
17	112.5	22.5	16.88
18	112.5	22.5	16.88
19	112.5	22.5	16.88
20	112.5	22.5	16.88

### Microbial strain

The strain *Schizochytrium* sp. I-F-9 (addressed as I-F-9 henceforth) was obtained by ARTP mutagenesis of *Schizochytrium* sp. (ATCC 20888) in our laboratory earlier. *Schizochytrium* sp. (ATCC 20888) was bought from the China Guangdong Microbial Culture Center and preserved in the Ruminant Nutrition laboratory (Institute of Animal Science, Chinese Academy of Agricultural Sciences). For cell preservation and transfer refer to the method of Zhao et al. (2017) [24].

### Fermentation condition

The seed culture medium consists of 30 g/L glucose, 10 g/L peptone, 5 g/L yeast extract, and 15 g/L sea salt. The initial fermentation medium consists of 100 g/L glucose, 5.6 g/L peptone, 20 g/L sodium glutamate, 2.5 g/L KH_2_PO4, 7.2 g/L MgSO_4_, 12.8 g/L Na_2_SO_4_, 0.4 g/L CaCl_2_, and 15 g/L sea salt. The medium was autoclaved at 115 °C for 30 min. The vitamin solution contained 0.1 g/L VB_1_, 0.1 g/L VB_6_, and 0.01 g/L VB_12_. All chemicals were purchased from Solarbio (Beijing, China), except for sea salt, which was purchased from Jiangxi Haiding Technology Company Limited (Jiangxi, China). It was filtered by a 0.22 micron filter and added to the medium. The stored cells were transferred into a 50-mL seeding medium (in 250-mL flasks) cultured for 48 h with 200 rpm stirring at 28 °C. After 48 h of seed broth culture, 10% v/v inoculum was injected into the initial fermentation medium that was incubated for 120 h at 28 °C with 200 rpm agitation (in 250-mL shake flask).

### Assay of dry cell weight

The I-F-9 growth was monitored using the dry cell weight (DCW). A sample of 30-mL fermentation broth was harvested every 24 h to test the DCW, total lipids and DHA production. In the cell growth curves test, all flasks were incubated in the same condition under 24, 48, 72, 96, 120, 144 and 168 h respectively. Three shake flasks were randomly selected to collect cells at the time of each sampling. The fermentation broth was put into a weighed 50-mL-tube and centrifuged at 8000 rpm for 15 min. The precipitate was then washed two times with double-distilled water and centrifuged twice, and the dry weight of the cells was measured after 24 h of freeze-drying. The DCW was calculated as follows:

$${\text{DCW}}\,\left( {\text{g/L}} \right) = \frac{{{\text{Freeze}}\,{\text{dried}}\,{\text{cell}}\,{\text{weight}}\,}}{{{\text{Fermentation}}\,{\text{broth}}\,{\text{volume}}\,\left( {\text{L}} \right)}}$$

### Total lipid extraction

The total lipid extraction was improved following the method by Zhao et al. [24]. The details were as follows: the freeze-drying powder were disrupted by incubating a mixture of 1 g freeze-dried powder and 8 mL of hydrochloric acid (6 mol/L) in a hot water bath at 65 °C for 1 h. The total fatty acid was extracted with 10 mL of n-hexane. Repeated the extraction three times, evaporating the n-hexane with a rotary nitrogen blower to harvest total lipids. The total lipid yield was calculated as follows:

$${\text{Total}}\,{\text{lipid}}\,{\text{yield}}\left( {\text{g/L}} \right) = \left( {{\text{Total}}\,{\text{lipid}}\,{\text{weight}}\,\left( {\text{g}} \right)} \right)/\left( { 1 \left( {\text{g}} \right)} \right) \times {\text{DCW}}\,\left( {\text{g/L}} \right)$$

### DHA yield and fatty acid analysis

Operate fatty acid methylation referring to the improved method of previous articles [25] as follows: 80 µL oil samples were added to tubes having 1 mL of 1 M KOH–methanol. The tubes were heated in a water bath at 65 °C for 30 min. After cooling the tube to indoor temperature, 2 mL of BF3–methanol was injected into it in a water bath at 65 °C for 30 min. When the tubes cooled to indoor temperature, 1 mL of n-hexane was injected to extract fatty acid methyl esters (FAMEs). The tubes were mixed through a vertex for 1 min, and then 1 mL of saturated sodium chloride was added to remove moisture from the tubes. The FAMEs samples were centrifuged at 3000 rpm for 2 min to separate the precipitate. The qualitative and quantitative the FAMEs are referenced from our previous study [26] using Agilent MassHunter Workstation Software (B.07.01, Agilent Technologies). The FAMEs were identified by comparing the retention times of methyl cis-4,7,10,13,16,19-DHA standard (CAS:301-01-9, Solarbio Beijing, China) and GLC NESTLE 37MIX (BYG8010, Solarbio, Beijing, China) (Additional file 1: Fig. S1). And then, the standard curves of the DHA standard were created based on the five different methyl-DHA content and corresponding peak areas (Additional file 1: Fig. S2).

## Results

### Effects of the different fermentation times and different carbon and nitrogen sources on fermentation

**Fig. 2:**
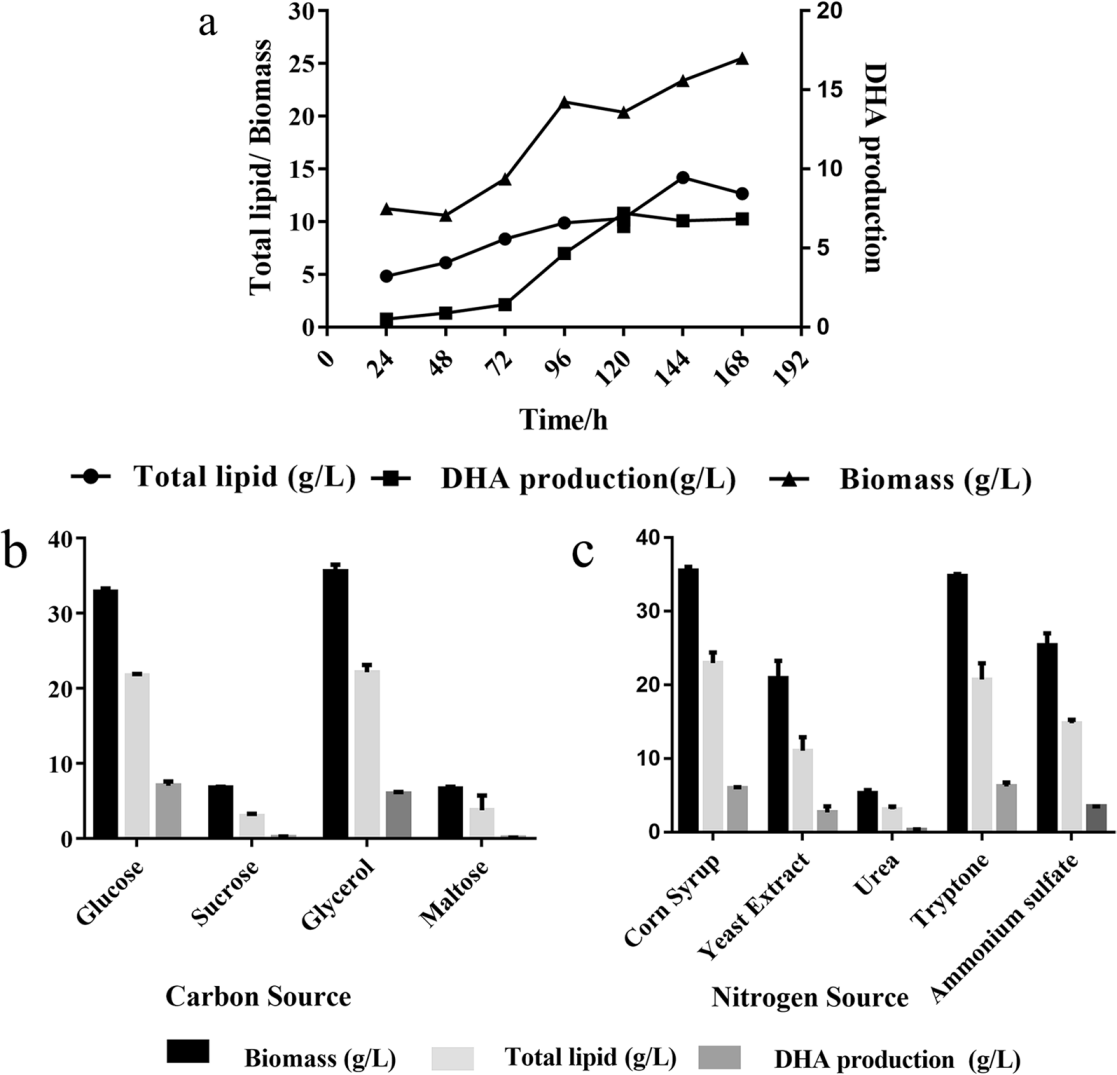
Biomass, total lipid, and DHA yields of I-F-9 at different fermentation times and with different carbon and nitrogen sources. **a** Effects of different fermentation time in biomass, total lipid and DHA production. **b** Fermentation medium containing 100 g/L glucose, sucrose, glycerol, and maltose as carbon sources and **c** Fermentation medium containing 10 g/L corn steep liquor, yeast extract, urea, peptone, and ammonium sulfate as nitrogen sources

As shown in Fig. 2a, the DHA yield remained constant at between 6.73 and 6.84 g/L even if the fermentation time increased from 120 to 168 h. From 24 to 168 h of fermentation, the highest fermentation efficiency was achieved at 56.51 ± 2.05 mg (L h)^−1^ in 120 h. Therefore, 120 h was determined the optimum fermentation time for I-F-9.

The overview of biomass, total lipid, and DHA yield of I-F-9, incubated in 250-mL shake flask supplying 50 mL of fermentation broths, were demonstrated in Fig. 2b, c. Different carbon and nitrogen sources greatly influenced the fermentation of I-F-9. In the fermentation broth of different carbon sources, the biomass of I-F-9 was between 6.64 g/L and 34.83 g/L, and the DHA production was between 0.10 and 7.00 g/L. Glucose and glycerol were significantly higher than the other treatments in I-F-9 growth and DHA accumulation with supplemented as a carbon source. The biomass was 34.83 g/L when glycerol was utilized as the carbon source. And when glucose was utilized as the carbon source, DHA production was 7.00 g/L. In addition, peptone was the best among fermentation supplemented by different nitrogen sources, where the biomass and DHA yields were 34.83 and 6.22 g/L, respectively.

### Screening significant growth parameters according to the plackett–burman design

The designed conditions for 12 tests and a data column (presenting the DHA production data of each test) are shown in Table 5. DHA production of I-F-9 varied between 4.551 and 8.443 g/L, depending on the culture parameters. The analysis of the data was done by the Design-Expert software. Relying on the software’s defaults, the factors with *P* ≤ 0.05 were considered the most influential, while the factors with *P* > 0.05 were considered less significant. Table 6 illustrates the results of significance levels of parameters highly correlated with the DHA concentration (*P* < 0.05), in which glucose (B), sodium glutamate (C), and sea salt (F) were suggested as the most significant parameters for further optimization.

**Table 5:** Results of the Plackett–Burman design

Run	A: peptone	B: glucose	C: sodium glutamate	D: MgSO_4_ 7H_2_O	E: KH_2_PO_4_	F: sea salt	DHA production (g/L)
1	1	1	− 1	1	1	1	6.665
2	− 1	1	1	− 1	1	1	6.416
3	1	− 1	1	1	− 1	1	7.005
4	− 1	1	− 1	1	1	− 1	6.489
5	− 1	− 1	1	− 1	1	1	7.685
6	− 1	− 1	− 1	1	− 1	1	8.443
7	1	− 1	− 1	− 1	1	− 1	6.417
8	1	1	− 1	− 1	− 1	1	7.297
9	1	1	1	− 1	− 1	− 1	5.31
10	− 1	1	1	1	− 1	− 1	4.551
11	1	− 1	1	1	1	− 1	6.274
12	− 1	− 1	− 1	− 1	− 1	− 1	7.330

**Table 6:** Significance (*P* values) of model and each variable using the Plackett–Burman design on the DHA production

Variable	Sum of squares	F-value	*P*-value
Model	10.52	7.03	**0.0246**
A: peptone	0.3156	1.26	0.3118
B: glucose	3.44	13.79	**0.0138**
C: sodium glutamate	2.43	9.74	**0.0262**
D: MgSO_4_ 7H_2_O	0.0881	0.353	0.5783
E: KH_2_PO_4_	8.33E−6	0	0.9956
F: sea salt	4.25	17.03	**0.0091**

*P* < 0.05 is marked in bold. When* P* < 0.05, the factor was considered as the most significant parameter to influence DHA production

### Optimization of medium components by CCD

The purpose of the CCD design was to identify the effect of different combinations of glucose, sodium glutamate and sea salt on the DHA production of I-F-9 cultured in a 250-mL shake flask. The results are displayed in Table 7. The DHA production of I-F-9 varied between 3.557 and 8.238 g/L depending on the incubation parameters (Table 7).

**Table 7:** Results of the central composite design

Run	A: glucose	B: sodium glutamate	C: sea salt	DHA production (g/L)	
				Actual	Predicted
1	− 1	− 1	− 1	7.535	7.309
2	1	− 1	− 1	8.105	8.015
3	− 1	1	− 1	3.557	3.582
4	1	1	− 1	3.582	3.824
5	− 1	− 1	1	6.270	5.662
6	1	− 1	1	6.239	5.848
7	− 1	1	1	6.537	6.262
8	1	1	1	6.124	5.984
9	− 1.682	0	0	5.976	6.444
10	1.682	0	0	6.756	6.805
11	0	− 1.682	0	5.989	6.595
12	0	1.682	0	3.665	3.576
13	0	0	− 1.682	5.960	5.813
14	0	0	1.682	5.579	6.244
15	0	0	0	7.177	7.598
16	0	0	0	7.256	7.598
17	0	0	0	7.269	7.598
18	0	0	0	8.238	7.598
19	0	0	0	7.870	7.598
20	0	0	0	7.866	7.598

The predicted values of DHA production utilizing the equation above and the data are given in Table 7. The experimental and predicted values of DHA yield were in good agreement. The corresponding analysis of variance (ANOVA) is given in Table 8. The regression model developed for DHA yield was significant (*P* = 0.0001); the lack-of-fit test demonstrated that the quadratic formula was the ideal model for data regression analysis (*P* = 0.2521 > 0.05) (Table 9). The regression equation R^2^ = 0.9598 indicated that the test results were plausible. The F test showed that the effects of B, BC, A^2^, B^2^, and C^2^ on DHA yield were significant; the effects of A, C, AB, and AC on DHA yield were insignificant (Table 8).

**Table 8:** Coefficients of the second-order polynomial model in 2^3^ central composite design

Factors	Coefficient	Standard error	F-value	*P*-value
Model			14.59	**0.0001**
Intercept	7.6	0.2141		
A-glucose	0.1071	0.1421	0.5688	0.4681
B-sodium glutamate	− 0.8976	0.1421	39.93	** < 0.0001**
C-sea salt	0.1281	0.1421	0.8132	0.3884
AB	− 0.1161	0.1856	0.3912	0.5457
AC	− 0.13	0.1856	0.4903	0.4997
BC	1.08	0.1856	33.96	**0.0002**
A^2^	− 0.3442	0.1383	6.19	**0.032**
B^2^	− 0.8882	0.1383	41.25	** < 0.0001**
C^2^	− 0.555	0.1383	16.11	**0.0025**

*P* < 0.05 is marked in bold. When* P* < 0.05, the factor was considered as the significant factor to influence DHA yield

**Table 9:** ANOVA for the central composite design (*R*^2^ = 0.9293; Adj *R*^2^ = 0.8656)

Source	Sum of squares	Degree of freedom	Mean square	F-value	*P*-value
Residual	2.76	10	0.2756		
Lack of fit	1.8	5	0.36	1.88	**0.2521**
Pure error	0.956	5	0.1912		
Cor total	38.95	19			

The lack-of-fit test is not significant at* P* > 0.05, which demonstrated that the quadratic formula was the ideal model for data regression analysis

The DHA yields were obtained from a series of CCD experiments, and were analyzed by regression utilizing a quadratic polynomial equation. The two regression formulas were indicated as coding factors and actual factors. Factors are expressed by capital letters: A: Glucose, B: Sodium glutamate, and C: Sea salt.

The final equation in terms of coded factors:

$$\begin{aligned} {\text{DHA}}\,{\text{yield}} & = 7.598 + 0.107139{\text{A}} - 0.897631{\text{B}} + 0.128103{\text{C}} - 0.116089{\text{AB}} \\ & - 0.129967{\text{AC}} + 1.08156{\text{BC}} - 0.344184{\text{A}}^{2} - 0.888183{\text{B}}^{2} - 0.554962{\text{C}}^{2} \\ \end{aligned}$$

The final equation in terms of actual factors:

$$\begin{aligned} {\text{DHA }}\,{\text{yield}} & = - 63.5387 + 0.681357{\text{A}} + 2.56018{\text{B}} + 0.828327{\text{C}} - 0.00371486{\text{AB}} \\ & \quad - 0.00554526{\text{AC}} + 0.230732{\text{BC}} - 0.00220278{\text{A}}^{2} - 0.142109{\text{B}}^{2} \\ & \quad - 0.157856{\text{C}}^{2} \\ \end{aligned}$$

**Fig. 3:**
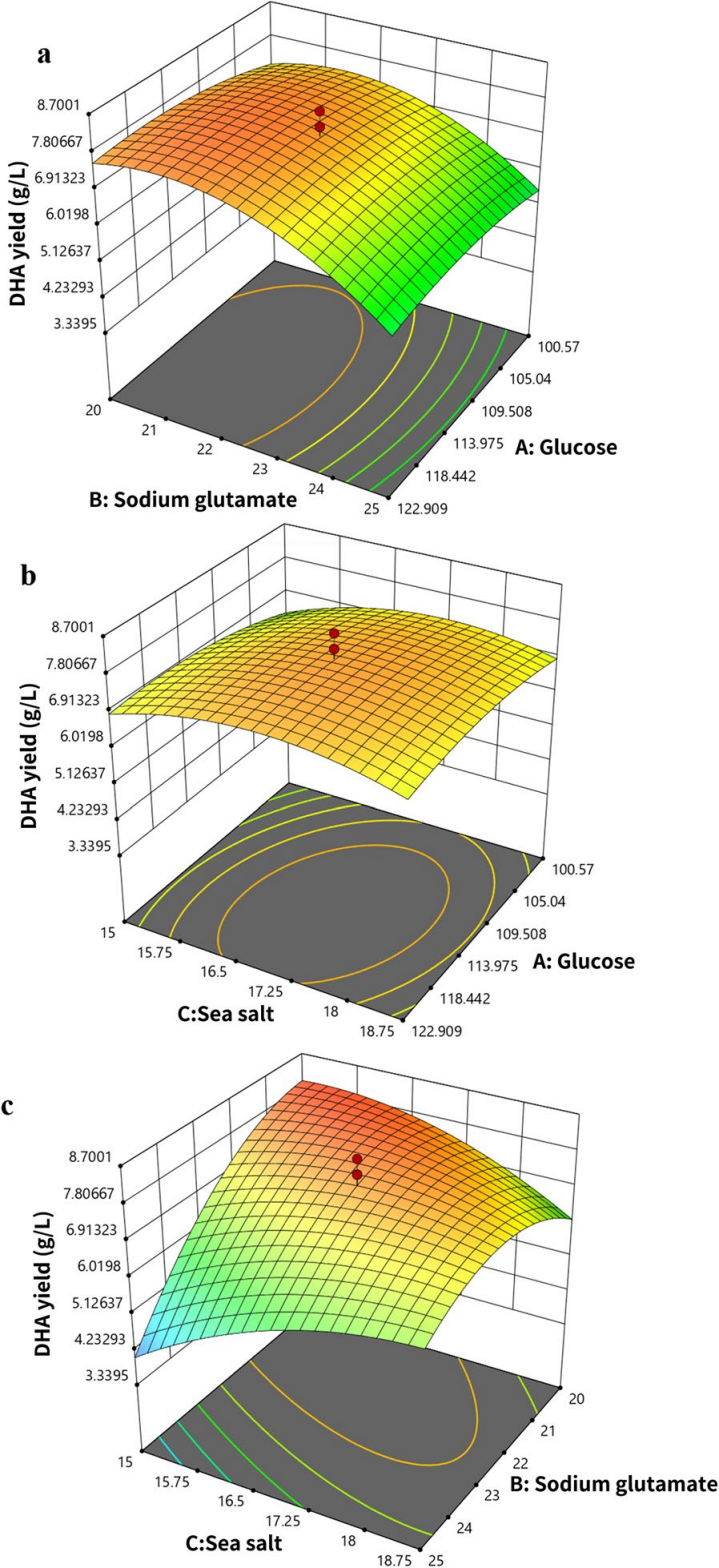
Contour plots depicting the response surface of DHA yield correlated to the levels of the variables: **a** glucose and sodium glutamate (C = 0); **b** glucose and sea salt (B = 0); and **c** sodium glutamate and sea salt (A = 0)

The regression analysis of the equations was performed utilizing the Design-Expert software (Design-Expert version 11.0.0) to obtain the model-optimized values of the medium components. Figure 3 shows the isoresponse contour lines of the medium components for optimized DHA production. The predicted optimal conditions were 118.71 g/L glucose, 20.00 g/L sodium glutamate, and 15.16 g/L sea salt.

### Cultivation on an optimized medium

The cells were cultured in 250-mL shake flask and fermented for 120 h at 28 °C and 200 rpm using 50 mL of the optimized medium to evaluate the growth and DHA production of I-F-9 in medium optimized by CCD. The experimental results of 120 h cultivation using an optimal medium containing 118.71 g/L glucose, 20.00 g/L sodium glutamate, and 15.16 g/L sea salt revealed that biomass, DHA concentration, and DHA productivity were 39.23 ± 0.56 g/L, 21.23% ± 0.038 and 69.41 ± 0.43 mg/L/h, respectively. The DHA and lipid production after fermentation was 8.33 ± 0.074 g/L and 30.24 ± 2.66 g/L, which was 34.73% and 19.34% greater than that prior to optimization (6.18 ± 0.09 g/L and 25.34 ± 2.11 g/L).

## Discussion

Several studies have confirmed the significant benefits of seafood with high contents of long-chain polyunsaturated fatty acids for human and animal health [27–29], in which DHA, a polyunsaturated fatty acid (PUFA), is considered as a major factor [30]. At present, the main sources of DHA on the market are fish oils and microbial lipids [21]. *Schizochytrium* sp. is a promising producer of microbial DHA, which has been considered the Generally Recognized as Safe (GRAS) status by the United States Food and Drug Administration [31]. Variations in fermentation parameters have a significant impact on DHA production from *Schizochytrium,* including carbon and nitrogen sources, management DO, the osmotic pressure of the medium, pH management and temperature control. As such, the production of DHA can be greatly enhanced by optimizing the medium formula and the fermentation process. Culture environment optimization can improve the production of DHA from *Schizochytrium*, such as intermittent oxygen treatment [32] and low-temperature hatch [33]. In terms of pH regulation, Zhao et al. found that *Schizochytrium* grew best in neutral conditions, while DHA synthesis increased under acidic conditions. Therefore, a two-stage pH control was developed to achieve a DHA yield of 11.44 g/L in *Schizochytrium* sp. AB-610 [24]. ARTP mutagenized *Schizochytrium* sp. I-F-9 used in this study contained higher oil content and DHA production than the original wild strain. If DHA production is to be further increased, then optimization of the nutrition conditions to obtain higher cell densities will be an important issue.

The CCD is a response surface methodology widely used for the fermentation optimization of various products, including food, beverages, and pharmaceuticals [34]. It describes the effects of interactions between parameters in linear and quadratic models. Here, the optimization of I-F-9 for the production of DHA was conducted as three phases, including the single-factor test to select the optimum carbon and nitrogen sources, following with the Plackett–Burman design to screen the major impacts of the variables and the response surface optimization.

The carbon and nitrogen sources in the medium have a significant effect on lipid synthesis in fungi [35]. This study showed that I-F-9 produced highest content of DHA with glucose and peptone as carbon and nitrogen source respectively, which was consistent with Bajpai's findings [36]. However, sucrose, maltose, urea, yeast extract and ammonium nitrate as carbon and nitrogen sources had limited effects on the cell growth of I-F-9, resulted in significantly lower DHA production than the others [37]. As a monosaccharide, glucose is an important carbon source in microbial fermentation [38]. Previous studies have shown that sugar can affect the growth and metabolism of microorganisms. For example, bilberry yeast grows better in glucose, while Komagataella grows better in fructose [39]. The DHA production in the glucose medium was higher than that in glycerol in this study. Acetyl-CoA is the main substrate for PUFA synthesis, which can be generated by glucose and glycerol. The stoichiometry of glucose metabolism is about 1.1 M of acetyl-CoA per 100 g. But approximately 1.1 M acetyl-CoA is generated by 110 g of glycerol metabolism [40, 41]. Therefore, glucose can produce more acetyl-CoA than glycerol, which might be the reason of the results mentioned above. Peptones are a complex nitrogen source, containing various nutrients, including proteins, peptides and free amino acids, as well as lower levels of carbohydrates, lipids, minerals, vitamins and growth factors. Besides improving the cell biomass, peptones can promote overall cell development, compared to other nitrogen sources [42, 43].

Glucose, sodium glutamate and sea salt were selected to have the greatest effects on DHA production of I-F-9 in Plackett–Burman design, which is in agreement with the findings of Manikan et al. [44]. Bajpai et al. [45] have confirmed that glucose concentration has not affected on the proportion of DHA in lipids but it can significantly affect cellular biomass and lipid content, and consequently DHA production. Ethier et al. [46] have reported that carbon sources can affect the biomass of *Schizochytrium* sp. and may influence PUFA synthesis. Sodium glutamate, a simple organic nitrogen source, is thought to promote biomass and increase the lipid yield of Thraustochytrids. Glutamate is usually found in the ocean as sodium salts (C_5_H_8_NNaO_4_), which is the main nonessential amino acid for marine organisms. Manikan et al. [44] have reported that the optimum sodium glutamate concentration can possibly result in higher DHA yields in *Aurantiochytrium*. And it is vital for any new strains to achieve significant levels of DHA production as an important nutrient [47]. Sodium glutamate causes high DHA production because it could regulate the activity of the enzyme of acetyl-CoA carboxylase [48] and glucose-6-phosphate dehydrogenase [49] that both can produce substrates for fatty acid synthesis (acetyl coenzyme A and NADPH). Besides the carbon and nitrogen sources, trace minerals can also affect the growth and lipid production of *Schizochytrium* as well as [50]. Here, sea salt containing a variety of essential minerals were used as the main trace element supplement to I-F-9.

Response surface design has been widely utilized to optimize many fermentation process parameters, including medium composition [34]. Most variations in response surface optimization can be interpreted by the regression equation [51]. In this study, the ANOVA of the DHA yield for I-F-9 showed that the F value was 14.59, which explained that the parameters in the model had a significant impact in the response experiment. In the model, the *P* value was 0.0001 demonstrating that the regression formula was statistically highly significant at the 95% confidence interval. Moreover, the lack-of-fit F value (1.88) means that the lack of fit was non-significant relative to the pure error. The R^2^ and Adj R^2^ for DHA yield were 0.9293 and 0.8656, respectively. Adequate precision estimates the signal-to-noise ratio, and a ratio higher than 4 is desirable [52]. The ratio of 11.958 illustrated an adequate signal.

To date, many articles have been carried out to elevate the DHA yield of *Schizochytrium* sp. utilizing various fermentation models and strategic techniques. Table 10 showed that the DHA yields of various Thraustochytrid strains growing in glucose and glycerol as the main carbon sources compared with I-F-9. Although higher DHA yields were obtained in other studies, the results were obtained at bioreactor scale [53, 54] or using two-stage control [24]. Manikan et al. [44] screened the optimum medium components through response surface methodology in shake flasks, which was then applied in a 5L bioreactor, showing that the biomass, total fatty acid and DHA production of *Aurantiochytrium* sp. SW1 were 17.8 g/L, 9.6 g/L, and 4.23 g/L in the shake flask and 24.46 g/L, 9.4 g/L and 4.5 g/L in the bioreactor, respectively. Hang et al. [32] screened glycerol concentrations of medium by shaking flasks for the first time and then used a 5-L bioreactor with intermittent oxygen control (maintaining a 50% DO level), which finally increased DHA production from 1.4 to 20.3 g/L. Accordingly, it is speculated that the DHA yield and content of I-F-9 obtained in the bioreactor with high cell density cultivation can be further improved. To enhance DHA yield of *Schizochytrium* sp. by optimization, Fu et al. [22] used low energy ion mutagenesis combined with the staining selection method and fermentation optimization. The results demonstrated that the yield and content of DHA was 6.52 g/L and 11.78%, respectively. The increase of DHA production in this study may be attributed to the increased concentration of glucose in the medium. Yokochi et al. [10] demonstrated that there might be the optimum glucose concentration to promote the growth of *Schizochytrium*. Hong et al. [54] carried out one-factor design for glucose optimization finally to elevated the DHA yield to 2.8 g/L, with DHA yield efficiency of 38.9 mg/L/h. In the present study, new strain I-F-9 obtained by ARTP mutagenesis in our lab was optimized by a more sophisticated CCD experimental design for the fermentation medium. The DHA yield of the strain I-F-9 was 8.33 ± 0.074 g/L with DHA productivity of 69.41 ± 0.43 mg (L h)^−1^, which was higher than our original culture condition (improved by 34.73%) and most of the previous studies.

**Table 10:** Summary of DHA production of various thraustochytrids compared with I-F-9

Strain	Carbon source	Nitrogen source	Culture time (h)	Device scale	DHA production (g/L)	DHA concentration (DHA/DCW, %)	References
*Schizochytrium* sp. S056	Glucose	Peptone, Yeast extract	144	250-mL	7.95	20.42	[61]
*Schizochytrium* sp. S31	Glycerol	Yeast extract, (NH4)_2_SO_4_	61/96	250-mL/50-L	6.53/28.93	–	[53]
*Schizochytrium* sp. S1	Glucose	Yeast extract, Sodium glutamate	168	500-mL	6.52	11.78	[22]
Aurantiochytriumsp. SW1	Glucose	Yeast extract, Sodium glutamate	96	250-mL/5-L	4.23/4.5	23.76/18.40	[44]
Aurantiochytrium limacinum SR21	Glycerol	Peptone, Yeast extract	168/165	500-mL/5-L	1.4/20.3	16.26/32.87	[32]
*Schizochytrium* sp. ATCC20888	Glucose	Yeast extract, Sodium glutamate	168	500-mL/50-L	7.12/12.89	–	[62]
*Schizochytrium* sp. AB-610	Glucose	Peptone, Sodium glutamate	120	250-mL	11.44	18.27	[24]
*Schizochytrium sp.* I-F-9	Glucose	Peptone, Sodium glutamate	120	250-mL	8.33	21.23	This study

Schizochytrium powder has been used in feed supplements in husbandry for decades [55]. Feeding lactating cows with Schizochytrium powder can improve the milk quality in dairy industry [56]. In goats, it can reduce methane production [57]. In lambs [58] and heifers [59], it can increase n-3 PUFA in the muscle. The concentrations of DHA in previous studies and our study has been summarized in Table 10. Xu et al. [60] have reported that the DHA content is over 10% of the dry matter in *Aurantiochytrium* sp. (*Schizochytrium* sp.), while 24% in fish oil. In this study, the optimized DHA concentration was 21.23%, which is better than most of the similar studies operated in shake flasks.

## Conclusions

In conclusion, the CCD method was applied to optimize the DHA yield by the second-order response surface model for the experimental data. The model prediction value was 8.10 g/L in DHA production. The optimized DHA yield of I-F-9 was 8.33 ± 0.074 g/L in the shake flask, which is essentially close to the predicted value. High DHA yields of *Schizochytrium* sp. could be obtained by the present method, which is potentially applicable for future production. The present study provides a fundamental basis to potentially use the *Schizochytrium* sp as the direct-fed microbials for animal and food industry.

## Supplementary Information

**Additional file 1.**. Chromatogram of DHA methyl ester standard and standard curves of the DHA methyl ester standard.

## Data Availability

The data that support the findings of this study are available from the corresponding author on reasonable request.

## References

[1] Whelan J, Rust C (2006). Innovative dietary sources of n-3 fatty acids. Annu Rev Nutr.

[2] Zhang MJ, Spite M (2012). Resolvins: anti-inflammatory and proresolving mediators derived from omega-3 polyunsaturated fatty acids. Annu Rev Nutr.

[3] Zhang TT (2019). Health benefits of dietary marine DHA/EPA-enriched glycerophospholipids. Prog Lipid Res.

[4] Li J (2021). Health benefits of docosahexaenoic acid and its bioavailability: a review. Food Sci Nutr.

[5] Salem N Jr, Eggersdorfer M. Is the world supply of omega-3 fatty acids adequate for optimal human nutrition? Curr Opin Clin Nutr Metab Care. 2015;18(2):147–54. 10.1097/MCO.0000000000000145 25635599

[6] Castejon N, Senorans FJ (2020). Enzymatic modification to produce health-promoting lipids from fish oil, algae and other new omega-3 sources: a review. N Biotechnol.

[7] Falk MC (2017). Developmental and reproductive toxicological evaluation of arachidonic acid (ARA)-Rich oil and docosahexaenoic acid (DHA)-Rich oil. Food Chem Toxicol.

[8] Wijendran V, Hayes KC (2004). Dietary n-6 and n-3 fatty acid balance and cardiovascular health. Annu Rev Nutr.

[9] Russo GL (2021). Sustainable production of food grade omega-3 oil using aquatic protists: reliability and future horizons. N Biotechnol.

[10] Yokochi T, Honda D, Higashihara T, Nakahara T (1998). Optimization of docosahexaenoic acid production by *Schizochytrium limacinum* SR21. Appl Microbiol Biotechnol.

[11] Darley WM, Porter D, Fuller MS (1973). Cell wall composition and synthesis via Golgi-directed scale formation in the marine eucaryote, *Schizochytrium aggregatum*, with a note on *Thraustochytrium* sp. Arch Mikrobiol.

[12] Heo S-W (2020). Application of Jerusalem artichoke and lipid-extracted algae hydrolysate for docosahexaenoic acid production by *Aurantiochytrium* sp. KRS101. J Appl Phycol.

[13] Chi G (2022). Production of polyunsaturated fatty acids by *Schizochytrium (Aurantiochytrium)* spp. Biotechnol Adv.

[14] Lewis KD (2016). Toxicological evaluation of arachidonic acid (ARA)-rich oil and docosahexaenoic acid (DHA)-rich oil. Food Chem Toxicol.

[15] Erkkila AT (2006). Higher plasma docosahexaenoic acid is associated with reduced progression of coronary atherosclerosis in women with CAD. J Lipid Res.

[16] Aasen IM (2016). Thraustochytrids as production organisms for docosahexaenoic acid (DHA), squalene, and carotenoids. Appl Microbiol Biotechnol.

[17] Du F (2021). Biotechnological production of lipid and terpenoid from thraustochytrids. Biotechnol Adv.

[18] Valentine REAM (2013). Single-cell oils as a source of omega-3 fatty acids an overview of recent advances. J Am Oil Chem Soc.

[19] Sukenik A, Wahnon R (1991). Biochemical quality of marine unicellular algae with special emphasis on lipid composition. I. *Isochrysis galbana*. Aquaculture.

[20] Molina Grima E (1992). EPA from Isochrysis galbana. Growth conditions and productivity. Process Biochem.

[21] Nazir Y (2018). Optimization of culture conditions for enhanced growth, lipid and docosahexaenoic acid (DHA) production of *Aurantiochytrium* SW1 by response surface methodology. Sci Rep.

[22] Fu J (2016). Enhancement of docosahexaenoic acid production by low-energy ion implantation coupled with screening method based on Sudan black B staining in *Schizochytrium* sp. Bioresour Technol.

[23] Zhao B (2018). Enhancement of Schizochytrium DHA synthesis by plasma mutagenesis aided with malonic acid and zeocin screening. Appl Microbiol Biotechnol.

[24] Zhao B (2017). Improvement of docosahexaenoic acid fermentation from *Schizochytrium* sp. AB-610 by staged pH control based on cell morphological changes. Eng Life Sci.

[25] Ren LJ (2009). Enhanced docosahexaenoic acid production by reinforcing acetyl-CoA and NADPH supply in *Schizochytrium* sp. HX-308. Bioprocess Biosyst Eng.

[26] Sun LL (2022). Odd- and branched-chain fatty acids in milk fat from Holstein dairy cows are influenced by physiological factors. Animal.

[27] Bos DJ (2016). Effects of omega-3 polyunsaturated fatty acids on human brain morphology and function: What is the evidence?. Eur Neuropsychopharmacol.

[28] Mallick R, Basak S, Duttaroy AK (2019). Docosahexaenoic acid,22:6n–3: its roles in the structure and function of the brain. Int J Dev Neurosci.

[29] Yu X (2019). Effects of the application of general anesthesia with propofol during the early stage of pregnancy on brain development and function of SD rat offspring and the intervention of DHA. Neurol Res.

[30] Swanson D, Block R, Mousa SA (2012). Omega-3 fatty acids EPA and DHA: health benefits throughout life. Adv Nutr.

[31] Ratledge C (2012). Omega-3 biotechnology: errors and omissions. Biotechnol Adv.

[32] Huang TY, Lu WC, Chu IM (2012). A fermentation strategy for producing docosahexaenoic acid in *Aurantiochytrium* limacinum SR21 and increasing C22:6 proportions in total fatty acid. Biores Technol.

[33] Hu F (2020). Low-temperature effects on docosahexaenoic acid biosynthesis in *Schizochytrium* sp. TIO01 and its proposed underlying mechanism. Biotechnol Biofuels.

[34] Pal D (2021). Optimization of medium composition to increase the expression of recombinant human interferon-beta using the Plackett–Burman and central composite design in *E. coli* SE1. 3 Biotech.

[35] Holdsworth JE, Ratledge C (1988). Lipid turnover in oleaginous yeasts. Microbiology.

[36] Bajpai P, Bajpai PK, Ward OP (1991). Production of docosahexaenoic acid by *Thraustochytrium aureum*. Appl Microbiol Biotechnol.

[37] Li ZY, Ward OP (1994). Production of *docosahexaenoic* acid by *Thraustochytrium roseum*. J Ind Microbiol.

[38] Wang Z (2021). Sugar profile regulates the microbial metabolic diversity in Chinese Baijiu fermentation. Int J Food Microbiol.

[39] Liu C (2019). Raw material regulates flavor formation via driving microbiota in Chinese liquor fermentation. Front Microbiol.

[40] Fakas S (2009). Evaluating renewable carbon sources as substrates for single cell oil production by *Cunninghamella echinulata* and *Mortierella isabellina*. Biomass Bioenerg.

[41] Polbrat T, Konkol D, Korczynski M (2021). Optimization of docosahexaenoic acid production by *Schizochytrium SP*.—a review. Biocatal Agric Biotechnol.

[42] Kujawska N (2021). Optimizing docosahexaenoic acid (DHA) production by *Schizochytrium* sp. grown on waste glycerol. Energies.

[43] Orak T (2018). Chicken feather peptone: a new alternative nitrogen source for pigment production by *Monascus purpureus*. J Biotechnol.

[44] Manikan V, Kalil MS, Hamid AA (2015). Response surface optimization of culture medium for enhanced docosahexaenoic acid production by a Malaysian thraustochytrid. Sci Rep.

[45] Bajpai P, Bajpai P, Ward O (1991). Optimization of production of docosahexaenoic acid (DHA) by*Thraustochytrium* aureum ATCC 34304. J Am Oil Chem Soc.

[46] Ethier S (2011). Continuous culture of the microalgae *Schizochytrium limacinum* on biodiesel-derived crude glycerol for producing docosahexaenoic acid. Bioresour Technol.

[47] Shene C (2010). Microbial oils and fatty acids: effect of carbon source on docosahexaenoic acid (C22: 6 n-3, DHA) production by thraustochytrid strains. J Soil Sci Plant Nutr.

[48] Kowluru A (2001). Activation of acetyl-CoA carboxylase by a glutamate- and magnesium-sensitive protein phosphatase in the islet beta-cell. Diabetes.

[49] Lan WZ, Qin WM, Yu LJ (2002). Effect of glutamate on arachidonic acid production from *Mortierella alpina*. Lett Appl Microbiol.

[50] Nagano N (2013). Effect of trace elements on growth of marine eukaryotes, tharaustochytrids. J Biosci Bioeng.

[51] Wu K (2018). Application of the response surface methodology to optimize the fermentation parameters for enhanced docosahexaenoic acid (DHA) production by *Thraustochytrium* sp. ATCC 26185. Molecules.

[52] Muthukumar M, Mohan D, Rajendran M (2003). Optimization of mix proportions of mineral aggregates using Box Behnken design of experiments. Cem Concr Compos.

[53] Chang G (2013). Fatty acid shifts and metabolic activity changes of *Schizochytrium* sp. S31 cultured on glycerol. Bioresour Technol.

[54] Hong WK (2011). Production of lipids containing high levels of docosahexaenoic acid by a newly isolated microalga, *Aurantiochytrium* sp. KRS101. Appl Biochem Biotechnol.

[55] Amorim ML (2021). Microalgae proteins: production, separation, isolation, quantification, and application in food and feed. Crit Rev Food Sci Nutr.

[56] Marques JA (2019). Increasing dietary levels of docosahexaenoic acid-rich microalgae: ruminal fermentation, animal performance, and milk fatty acid profile of mid-lactating dairy cows. J Dairy Sci.

[57] Mavrommatis A (2021). Alterations in the rumen particle-associated microbiota of goats in response to dietary supplementation levels of *Schizochytrium* spp. Sustainability.

[58] Diaz MT (2017). Feeding microalgae increases omega 3 fatty acids of fat deposits and muscles in light lambs. J Food Compos Anal.

[59] Rodriguez-Herrera M (2018). Feeding microalgae at a high level to finishing heifers increases the long-chain n-3 fatty acid composition of beef with only small effects on the sensory quality. Int J Food Sci Technol.

[60] Xu XD (2020). The strategies to reduce cost and improve productivity in DHA production by *Aurantiochytrium* sp.: From biochemical to genetic respects. Appl Microbiol Biotechnol.

[61] Chen W (2016). Improvement in the docosahexaenoic acid production of *Schizochytrium* sp. S056 by replacement of sea salt. Bioprocess Biosyst Eng.

[62] Lin Y (2018). Optimization of enzymatic cell disruption for improving lipid extraction from *Schizochytrium* sp. through response surface methodology. J Oleo Sci.

