# Supplementary material for: Enhancing docosahexaenoic acid production of Schizochytrium sp. by optimizing fermentation using central composite design
Source: BMC Biotechnol. 2022 Dec 9;22:39. doi: 10.1186/s12896-022-00769-z (PMC9737722; doi:10.1186/s12896-022-00769-z)

**Supplementary materials**

Enhancing docosahexaenoic acid production of *Schizochytrium* sp. by optimizing fermentation using central composite design

**Jun Ding^1^, Zilin Fu^1^, Yingkun Zhu^1^, Junhao He^1^, Lu Ma^1^* and Dengpan Bu^1^***

^1 State Key Laboratory of Animal Nutrition, Institute of Animal Sciences, Chinese Academy of Agricultural Sciences, Beijing, China^

^*Corresponding author: Dengpan Bu and Lu Ma. State Key Laboratory of Animal Nutrition, Institute of Animal Science, Chinese Academy of Agricultural Sciences, No. 2 Yuanmingyuan West Road, Beijing, 100193, China. (E-mail: budengpan@126.com; malu.nmg@163.com; Tel: 86-10-62813901)^

Supplementary Figure S1. Chromatogram of fatty acid methyl ester: (a) Chromatogram of DHA methyl ester standard. (b) Chromatogram of fatty acid methyl ester in sample from I-F-9.
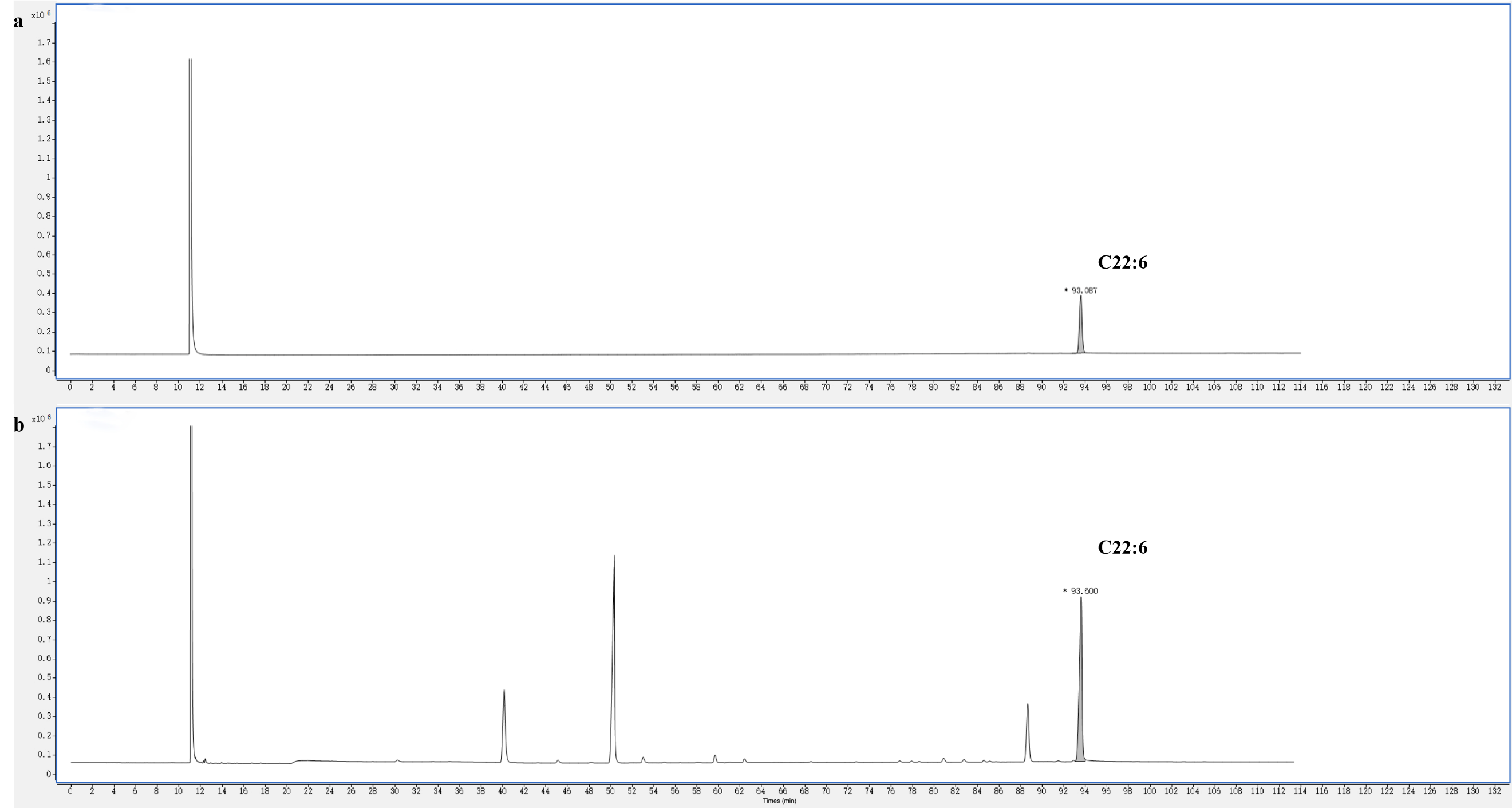


Supplementary Figure S2. Standard curves of DHA methyl ester.
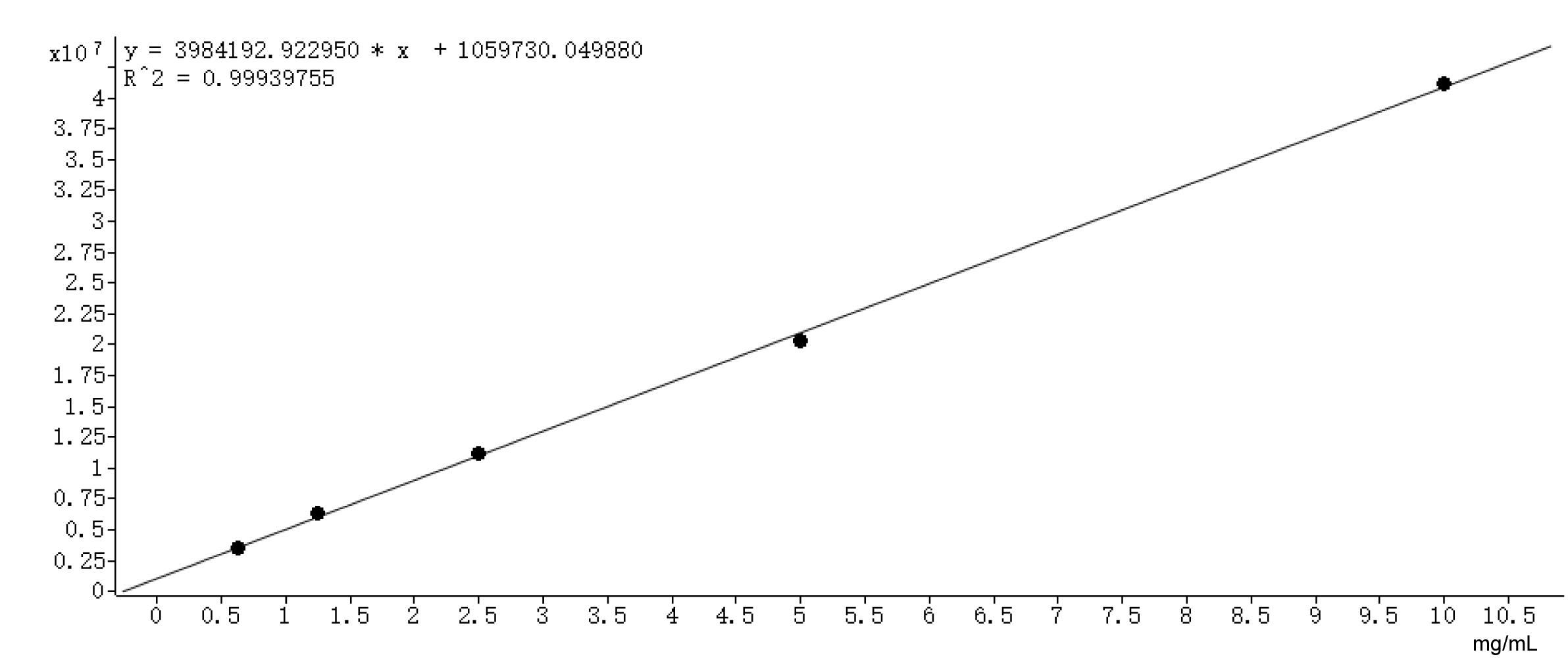

Supplement: Supplementary file 1 — Additional file 1.. Chromatogram of DHA methyl ester standard and standard curves of the DHA methyl ester standard. [file 12896_2022_769_MOESM1_ESM.docx]
